# Comparison of Mechanical Properties and Energy Absorption of Sheet-Based and Strut-Based Gyroid Cellular Structures with Graded Densities

**DOI:** 10.3390/ma12132183

**Published:** 2019-07-07

**Authors:** Dawei Li, Wenhe Liao, Ning Dai, Yi Min Xie

**Affiliations:** 1 College of Mechanical and Electrical Engineering, Nanjing University of Aeronautics and Astronautics, Nanjing 210016, China; 2 Center for Innovative Structures and Materials, School of Engineering, RMIT University, GPO Box 2476, Melbourne, VIC 3001, Australia

**Keywords:** gyroid-based cellular structure, functionally graded structures, mechanical properties, energy absorption

## Abstract

Bio-inspired functionally graded cellular materials (FGCM) have improved performance in energy absorption compared with a uniform cellular material (UCM). In this work, sheet-based and strut-based gyroid cellular structures with graded densities are designed and manufactured by stereo-lithography (SLA). For comparison, uniform structures are also designed and manufactured, and the graded structures are generated with different gradients. The mechanical behaviors of these structures under compressive loads are investigated. Furthermore, the anisotropy and effective elastic modulus of sheet-based and strut-based unit gyroid cellular structures are estimated by a numerical homogenization method. On the one hand, it is found from the numerical results that the sheet-based gyroid tends to be isotropic, and the elastic modulus of sheet-based gyroid is larger than the strut-based gyroid at the same volume fraction. On the other hand, the graded cellular structure has novel deformation and mechanical behavior. The uniform structure exhibits overall deformation and collapse behavior, whereas the graded cellular structure shows layer-by-layer deformation and collapse behavior. Furthermore, the uniform sheet-based gyroid is not only stiffer but also better in energy absorption capacity than the uniform strut-based gyroid structure. Moreover, the graded cellular structures have better energy absorption capacity than the uniform structures. These significant findings indicate that sheet-based gyroid cellular structure with graded densities have potential applications in various industrial applications, such as in crashworthiness.

## 1. Introduction

Functionally graded cellular materials (FGCM) are widely found in nature, have a relatively low-density with high strength, excellent energy absorption, and thermal conductivity [1]. Traditionally, cellular materials are formed during bulk processing, such as foaming to obtain random foam materials or to establish lattice structures by processes such as adhesion, welding, and extrusion [2]. However, these methods are difficult to obtain a graded cellular material, while variable density graded cellular materials have been shown to have better mechanical properties and energy absorption capabilities [3]. Recently, additive manufacturing (AM) techniques have made it possible to manufacture complex graded materials and structures, which make them potentially useful in biomedical, aerospace, and automotive applications [4]. 

Energy absorption performance of cellular materials fabricated by AM has become a hot research topic in recent years. Typically, additively manufactured honeycomb structures have been widely used in impact protection and energy absorption applications [5,6,7,8,9]. For instance, Correa et al. [5] studied the energy absorption capability of 3D printed negative stiffness honeycombs using selective laser sintering (SLS) technology with nylon material. According to the research results, the energy absorption capacity of the optimized negative stiffness honeycomb was comparable to that of the conventional honeycomb structure but has the advantages of being recyclable and reusable. In addition, Bates et al. [6] used a fused filament fabrication (FFF) 3D printing of two types of thermoplastic polyurethane (TPU) of hyper-elastic honeycomb structures with different relative densities. Then the in-plane energy absorption capacity of the structure was also studied. Besides, Habib et al. [7] studied the in-plane static compression fracture behavior and energy absorption capacity of 3D printed polymer honeycomb structures with different wall thicknesses. It was found that the experimental results obtained are consistent with the theoretical model for calculating the numerical solution and the wall thickness to length ratio, so the energy absorption of the honeycomb structures of different shapes is predictable. Since the 2D honeycomb structure exhibits strong anisotropy in three main directions, the orthotropic cellular material in three-dimensional space is more valuable. Instead, Mohsenizadeh et al. [8] studied the energy absorption properties of a large-scale octet-based cellular structure fabricated by photocuring under reciprocating loading and revealed that the structure could recover its original shape after compression to 70% strain, and the structure energy absorption efficiency is 11% higher than aluminum foam. Besides, Habib et al. [9] studied the energy absorption and deformation behavior of polymer lattice structures of six different unit cell topologies fabricated by Multi Jet Fusion (MJF) technology. It has been found that the lattice structure with bending-dominated deformation has lower stiffness and strength, but good energy absorption capacity. Stretch and buckling dominated structures are stronger and stiffer but have lower energy absorption properties. The discussion above is the energy absorption of uniform cellular materials.

For functionally graded cellular structures (FGCS), they have novel deformation and mechanical behaviors. Moreover, the potential for improved energy absorption has also been shown in practical studies [10,11,12,13,14,15]. For example, Bates et al. [10] first studied the effect of different gradient gradings produced by TPU materials on the energy absorption of honeycomb structures. The results show that the energy absorption curves of these structures can be controlled by classifying the density of the structures. Furthermore, the effects of cyclic loading and impact loading on energy absorption of gradient grading honeycomb structures were studied [11]. The results show that the gradient grading structure has excellent impact protection ability under extreme environmental conditions. Besides, Maskery et al. [12,13] fabricated and performed compression experiments on a uniform and graded BCC (body-centered cubic) lattice structures using SLS nylon [12] and SLM (selective laser melting) Al-Si10-Mg materials [13], respectively. The results demonstrate that the energy absorption capacity of the graded cellular materials of the two process conditions were superior to that of the uniform cellular materials. Additionally, Choy et al. [14] also used the SLM process to fabricate the graded lattice structure of two topologies and perform compression test analysis. The results show that the energy absorption effect of the graded lattice structure was far better than the uniform structure. Similarly, Al-Saedi et al. [15] used SLM technology to fabricate the graded F2BCC lattice structure of Al-12Si material and carried out compression tests. The results also show that the total cumulative energy absorption per unit volume was higher in functionally graded lattice than in uniform lattice. Therefore, the above studies show that the graded structure has better energy absorption capacity than the uniform structure.

To date, the above FGCS design has been basically assembled using truss-based cells. These pillar-based geometries have lower manufacturability [16]. Moreover, these lattice structures are prone to stress concentration during loading, resulting in structural failure [17,18,19]. However, lattice materials based on triply periodic minimal surfaces (TPMS) have received extensive attention in recent years. Not only does it have a smooth surface, it is not prone to stress concentration, and the function-driven variable-density cellular material is easily controlled to generate [20]. Li et al. [21] proposed the design method of variable-density gyroid cellular structure earlier and effectively used to optimize the stiffness of the object. Maskery et al. [22] evaluated the energy absorption capacity of a uniform sheet-based gyroid based on different scales of Al-Si10-Mg materials made by SLM technology. Abueidda et al. [23] performed experimental analysis on mechanical properties and energy absorbing capabilities using different sheet-based TPMS with uniform structures printed on SLS nylon. Similarly, Zhang et al. [24] performed manufacturing and compression analysis of a 316L stainless steel sheet-based TPMS lattice structure based on SLM. The above results show that the uniform sheet-based TPMS lattice structure has better energy absorption capacity. Recently, Yang et al. [25] used the SLM process to fabricate a graded strut-based gyroid structure and perform compression analysis. The results show that the graded gyroid structure has better energy absorption capacity in the horizontal compression direction than uniform gyroid structure.

In this work, strut-based and sheet-based gyroid cellular structures with uniform and graded densities are designed and fabricated by stereo-lithography (SLA) technique. The density of the uniform gyroid structure is set as 0.3, and the densities of graded gyroid structure are linear changing and set as 0.2 to 0.4 and 0.1 to 0.5, respectively, then the average density also is 0.3. Finally, each object is subjected to a compression test, and its deformation, mechanical behavior, and energy absorption capacity are analyzed. Besides, the numerical homogenization method is used to evaluate the anisotropy properties of the two types of gyroid-based cellular structures.

## 2. Materials and Methods

### 2.1. The Gibson-Ashby Model of Cellular Structures

**Figure 1:**
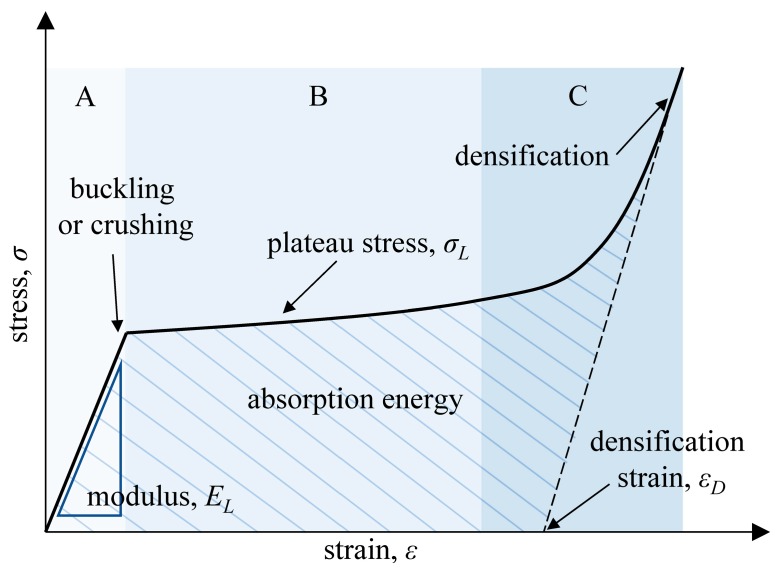
Characteristic compressive stress-strain curve of a cellular structure.

Gibson and Ashby et al. [26] systematically demonstrate the relationship between the mechanical properties of the cellular structure and the relative density, such as Young’s modulus, plateau stress, and densification strain (in Figure 1). These relationships are known as the Gibson-Ashby model:

(1) $$\frac{E_{L}}{E_{S}}=C_{1}{\left(\frac{\rho_{L}}{\rho_{S}}\right)}^{n}$$

(2) $$\frac{\sigma_{L}}{\sigma_{S}}=C_{2}{\left(\frac{\rho_{L}}{\rho_{S}}\right)}^{m}$$

(3) $$\varepsilon_{D}=1-\alpha \left(\frac{\rho_{L}}{\rho_{S}}\right)$$

where *E_L_*, *ρ_L_*, and *σ_L_* are the elastic modulus, density, and yield strength of cellular structures, respectively; *E_S_*, *ρ_S_*, and *σ_S_* are the elastic modulus, density, and yield strength of base solid material, respectively. *ε_D_* is the value of onset densification strain of the cellular structure where the densification region starts. Moreover, *ε_D_* is considered to be the practical limit for energy absorption applications using cellular structures. After this strain limit, the cellular structure will continue to absorb energy, but at the expense of transferring stress throughout the cellular structure. This can result in damage to the objects or injury to the human body. Moreover, the constant parameters *C*_1_, *C*_2_, *n*, *m*, and *α* are calculated by fitting the result of the compression test. In this work, numerical integration is used to determine the energy absorption capacity of the cellular structure, giving the area under the compressive stress-strain curves [27]. Then, the energy absorption per unit volume *W_v_* is expressed as:

(4) $$W_{v}=\int_{0}^{\varepsilon }\sigma (\varepsilon )d\varepsilon$$

### 2.2. Design of Gyroid-Based Cellular Structures

**Figure 2:**
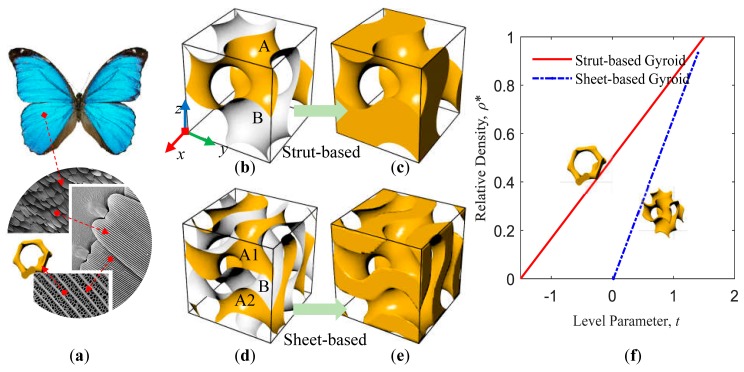
(**a**) Gyroid structure derived from bionic butterfly wings; gyroid surface (**b**) close to be strut-based gyroid structure (**c**) and double gyroid surface (**d**) close to be sheet-based gyroid structure (**e**); (**f**) is the relationship between relative density and parameter *t* of the two types gyroid cellular structures.

The gyroid surface is one of the most common types of triply periodic minimal surfaces, which was discovered in the 1970s by NASA (National Aeronautics and Space Administration) scientist Alan Schoen and designed for lightweight high-strength new materials [28]. Besides, the gyroid structure has been found to be steadily present in nature, such as the microstructure of butterfly wings [29] (as shown in Figure 2a). Moreover, the gyroid cellular structure has three advantages: (1) The structure is connectable and can easy exclude powder or liquid resin when manufactured by AM. (2) Its spiral structure has a self-supporting capability during the manufacturing process by AM. (3) Its structure has a high specific strength at low density [30]. Furthermore, the gyroid structure has a wider connection area than other typical TPMS structures (such as Schwarz Diamond and Primitive structures), which is advantageous for generating graded cellular materials. 

The TPMS equation describes a three-dimensional (3D) surface. To satisfy AM, we close the boundary to form a void-solid cellular material. Particularly, the equivalent parameter *t* is introduced into the original minimal surface equation, so that it can obtain gyroid cellular structures of different densities. In this work, we designed two types of gyroid cellular structures, which close the space based on the gyroid surface and the double gyroid surface. The triply periodic level surface functions of the two types of surfaces are:

(5) $$F_{G}(x,y,z)=\sin\left(2\pi \frac{x}{L}\right)\times \cos\left(2\pi \frac{y}{L}\right)=\sin\left(2\pi \frac{y}{L}\right)\times \cos\left(2\pi \frac{z}{L}\right)=\sin\left(2\pi \frac{z}{L}\right)\times \cos\left(2\pi \frac{x}{L}\right)-t$$

(6) $$F_{DG}(x,y,z)={\left[\sin\left(2\pi \frac{x}{L}\right)\times \cos\left(2\pi \frac{y}{L}\right)=\sin\left(2\pi \frac{y}{L}\right)\times \cos\left(2\pi \frac{z}{L}\right)=\sin\left(2\pi \frac{z}{L}\right)\times \cos\left(2\pi \frac{x}{L}\right)\right]}^{2}-t^{2}$$

where Equation (5) is the gyroid surface (Figure 2b) and Equation (6) is the double gyroid surface (Figure 2d), and *L* is the cubic unit cell edge length, and the level parameter *t* is a variable and determines the volume fractions pertaining to the regions separated by the surface. As shown in Figure 2c, the cellular structure obtained based on the gyroid surface is called as a strut-based gyroid cellular structure. Moreover, as shown in Figure 2e, the cellular structure obtained based on the double gyroid surface is called a sheet-based gyroid cellular structure. In this work, by sampling the volume fraction of the cellular structure under different level parameters *t*, and then fitting the relationship between the parameter *t* and the relative density ρ* of the gyroid cellular structure, the equations are as follows:

(7) $$\rho_{\mathrm{Struct}-G}^{*}(t)=0.333\times t+0.501\;-1.5<t<1.5$$

(8) $$\rho_{\mathrm{Sheet}-G}^{*}(t)=0.675\times t+0.012\;0.018<t<1.5$$

It can be seen from the results that the relative density ρ* is linear with the level parameter *t* (as shown in Figure 2f). Therefore, it allows precise control of the volume fraction of the graded gyroid cellular structures. Also, it is easy to obtain uniform and gradient-changing gyroid structures. 

**Figure 3:**
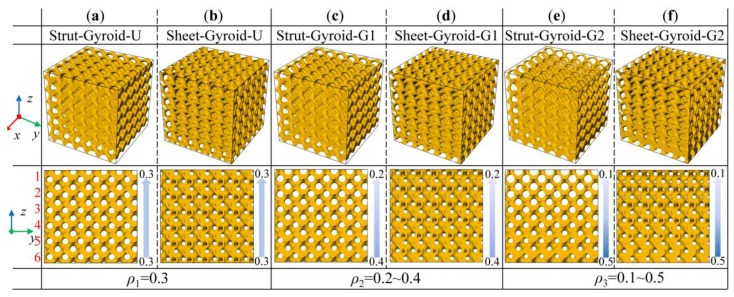
3D models of the (**a**) and (**b**) are the uniform gyroid-based cellular structures; (**c**) and (**d**) are the graded gyroid-based cellular structure structures with gradient-1 and (**e**) and (**f**) with gradient-2.

In this work, six sets of models are designed for strut-based and sheet-based gyroid cellular structure for experimental analysis. The design area is a cubic area of 36 × 36 × 36 mm, and the size of the cellular unit is set as 6 × 6 × 6 mm. Their average volume fraction is 0.3, with the uniform structure with a volume fraction of 0.3, the gyroid cellular structure that linearly changes the density from 0.2 to 0.4, called gradient-1, and the gyroid cellular structure that linearly changes the density from 0.1 to 0.5, called gradient-2. As shown in Figure 3, the cellular structures with smooth and continuously varying densities can be obtained, and the corresponding nomenclature of these cellular structures can be found in the figure. 

### 2.3. Manufacturing the Cellular Specimens

**Figure 4:**
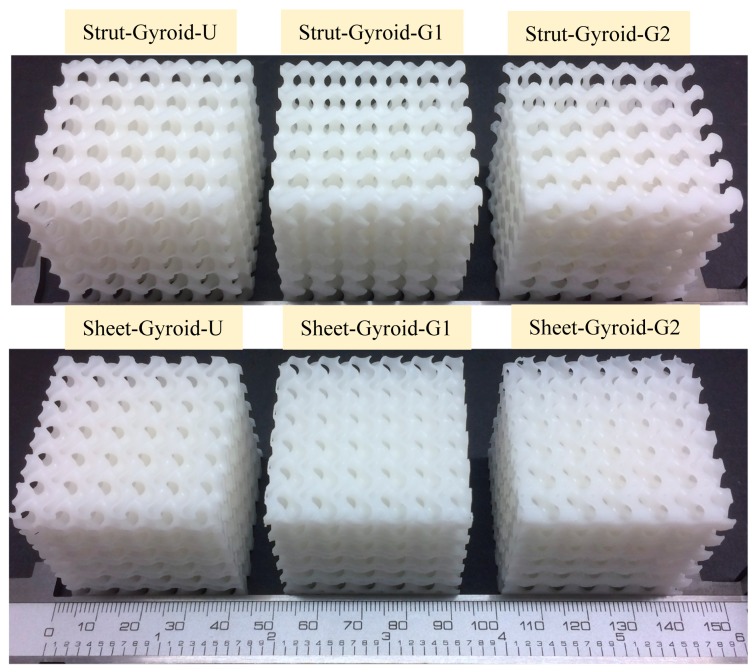
The fabricated gyroid uniform and graded cellular structures with different design parameters.

In this work, the designed uniform and graded gyroid-based cellular specimens are fabricated through SLA technology using a Form 2 (Formlabs Inc., Somerville, MA, USA) desktop 3D printer. SLA 3D printing technology can obtain a smooth cellular structure, which can avoid the impact of mechanical properties evaluation due to defects. First, the designed cellular specimens were imported into the Formlabs pre-processing software. Then, all models were printed in the same direction as the compression experiment and were fabricated using the same layer thickness and exposure time. This ensures consistency in the rest of the experiment. Additionally, each type of object was fabricated for three for repeated experimental analysis. Also, the manufactured samples are presented in Figure 4. 

### 2.4. Numerical Homogenization

In many applications, the anisotropy of structure is considered to be harmful, especially when the anisotropic cellular structures are used as structural components or energy absorption materials. Theoretically, the anisotropic structure is the worst in its weakest direction due to the uncertain load, and the anisotropic structure is prone to buckling failure. Therefore, this study will analyze the anisotropy of the two types of gyroid structures and investigate whether they affect energy absorption.

In this work, a simple and straightforward numerical homogenization method [31,32] is used to calculate the effective stiffness matrix and anisotropy analysis of strut-based and sheet-based gyroid cellular structures with different densities. Moreover, it can be used to verify the relative accuracy of compression experiments. For a given unit cellular structure, noting its homogenized elasticity tensor as $C_{ijkl}^{H}$, and according to Hooke’s law, $\sigma_{ij}=C_{ijkl}^{H}\varepsilon_{kl}$, where $\sigma_{ij}$ and $\varepsilon_{kl}$ are the effective stress and strain tensor, respectively. In the numerical implementation [33], in each step, one strain component is set to one, and the remaining five are zero, e.g., Equation (9).

(9) $$\mathrm{Input}:\;\left\{\begin{array}{l} \varepsilon_{11}\\ \varepsilon_{22}\\ \varepsilon_{33}\\ \varepsilon_{23}\\ \varepsilon_{31}\\ \varepsilon_{12} \end{array}\right\}=\left\{\begin{array}{l} 1\\ 0\\ 0\\ 0\\ 0\\ 0 \end{array}\right\}\;\mathrm{Output}:\;\left\{\begin{array}{l} \sigma_{11}\\ \sigma_{22}\\ \sigma_{33}\\ \sigma_{23}\\ \sigma_{31}\\ \sigma_{12} \end{array}\right\}=\left\{\begin{array}{l} C_{11}\\ C_{21}\\ C_{31}\\ C_{41}\\ C_{51}\\ C_{61} \end{array}\right\}$$

During this process, the unit strain is expressed as the specified displacement on the boundary. Therefore, the corresponding stress can be calculated by the reaction forces in the FEA. Then, the stiffness matrix of the gyroid-based cellular structure is calculated by six FEA. Besides, the computation process is realized by the Python language with ABAQUS. According to the calculation results, the gyroid structure belongs to the cubic symmetric system. Therefore, in the stiffness matrix: *C*_11_ = *C*_22_ = *C*_33_, *C*_12_ = *C*_13_ = *C*_23_, *C*_44_ = *C*_55_ = *C*_66_ and other constants are zero. Then, the expression form of the stiffness matrix is:

(10) $$\begin{array}{l} C_{G}^{H}=\left[\begin{array}{cccccc} C_{11} & C_{12} & C_{12} & 0 & 0 & 0\\ C_{12} & C_{11} & C_{12} & 0 & 0 & 0\\ C_{12} & C_{12} & C_{11} & 0 & 0 & 0\\ 0 & 0 & 0 & C_{44} & 0 & 0\\ 0 & 0 & 0 & 0 & C_{44} & 0\\ 0 & 0 & 0 & 0 & 0 & C_{44} \end{array}\right] \end{array}$$

According to Equation (10), the (axial) Young’s moduli *E^H^*, (axial) Poisson’s ratio *υ^H^*, and (axial) shear modulus *G^H^* can be calculated using tensor components. Moreover, we can use Zener [34] ration *A^H^* to measure the isotropy of cellular material, and if the value is close to 1, it means the cellular structure is isotropic.

(11) $$\begin{array}{l} E^{H}=\left({\left(C_{11}\right)}^{2}+C_{12}C_{11}-2{\left(C_{12}\right)}^{2}\right)/\left(C_{11}+C_{12}\right)\\ \upsilon^{H}=C_{12}/\left(C_{11}+C_{12}\right)\\ G^{H}=C_{44}\\ A^{H}=2C_{44}/\left(C_{11}-C_{12}\right) \end{array}$$

Therefore, the above numerical analysis method was used for the anisotropic analysis of the gyroid structures in Section 3.1. 

### 2.5. Mechanical Testing

Mechanical compression tests were performed to extract the mechanical behaviors of designed specimens. All samples were subjected to compression analysis using a TestResources 313 universal tester (USA) at room temperature with a 10 kN load cell with the crosshead loading rate to 10 mm/min, following the ASTM D695-15 standard [35]. Besides, the specimens were treated with UV curing at the same time to ensure that the model is fully cured. For uniform gyroid cellular structures, select the manufacturing direction for compression experiments. For the graded gyroid cellular structures, the gradient direction was selected for compression experiments, and the region with a small density was used as the top, and the dense one was used as the bottom. Moreover, the video compression method was used to record the compression deformation process. Additionally, to characterize the properties of the base material, three types of tensile standard specimens have dimensions as described in the standard test method for tensile properties of plastics (ASTM D638-14). Then, the tested base material elastic modulus is *E_S_* = 850.1 ± 15.2 MPa, yield stress *σ_S_* = 132.5 ± 12.8 MPa Poisson’s ratio is 0.33. 

## 3. Results and Discussion

### 3.1. Formatting of Mathematical Components

**Figure 5:**
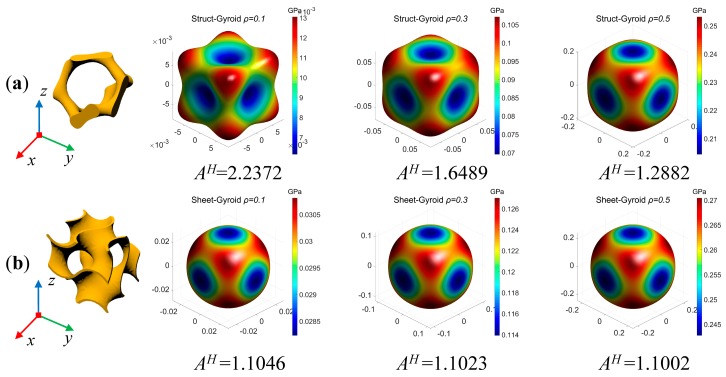
Anisotropy of strut-based gyroid (**a**) and sheet-based gyroid (**b**). (If *A^H^* is close to unity, the structure could be treated as isotropic).

As shown in Figure 5, the anisotropy of density varies with the strut-based gyroid cellular structures and the sheet-based gyroid cellular structures. To visually express the anisotropy of the two cellular structures, Young’s modulus surface is used to plot the elastic modulus in any direction of the 3D space, and the calculation is programmed in MATLAB. Therefore, the strong and weak directions can be clearly demonstrated. The elastic anisotropy of the cellular structure is highly dependent on the spatial arrangement of the unit materials. Although the gyroid cellular structure is not easy to see symmetry in geometry, it can be seen from the analysis of the anisotropic that it belongs to the form of the cubic crystal system. Moreover, it can be seen from the results that the sheet-based gyroid cellular structure is similar to isotropic at both low-density and high-density, while the strut-based gyroid structure has obvious anisotropy. Additionally, the sheet-based gyroid structure has a higher Young’s modulus than the strut-based gyroid structure at the same density. In theory, anisotropic structures are not conducive to energy absorption. For example, in an automobile under uncertain load conditions, the energy absorption in the weak direction of the structure will be weak. Moreover, the mechanical behaviors and energy absorption capacity of the two types of gyroid-based cellular structures will be compared in the following sections. 

### 3.2. Deformation of Uniform Gyroid-Based Cellular Structure

**Figure 6:**
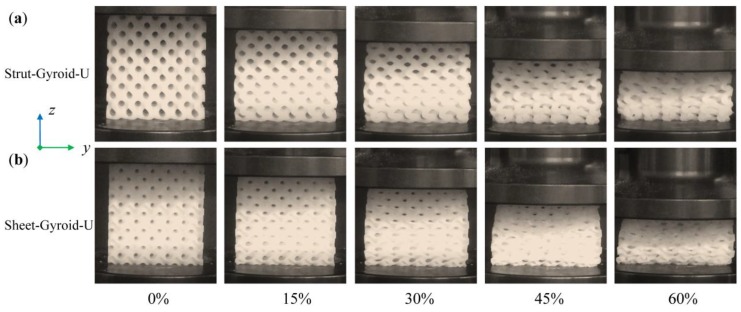
Deformation stages of Strut-Gyroid-U (**a**) and Sheet-Gyroid-U; (**b**) cellular structures in the presence of 0%, 15%, 30%, 45%, and 60% compression strain from the video capture.

**Figure 7:**
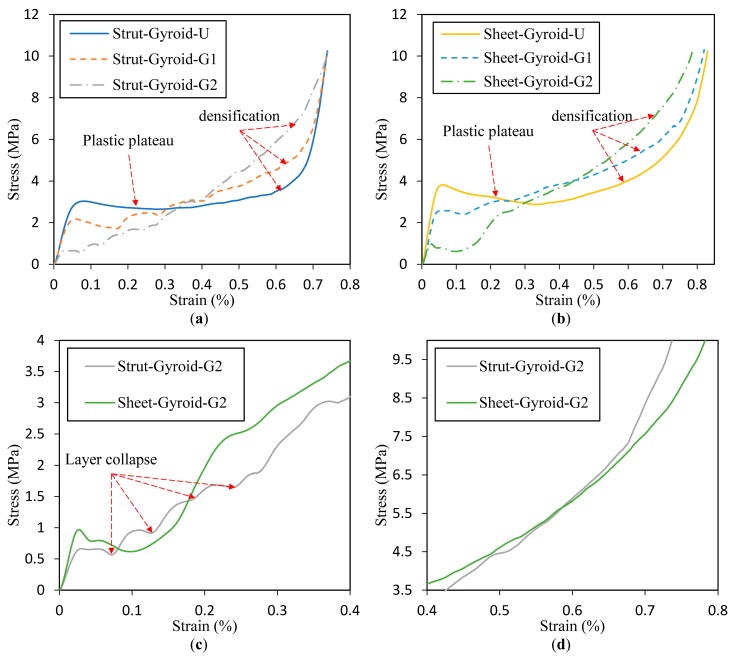
Compressive stress-strain curves of strut-based (**a**,**c**) and sheet-based (**b**,**d**) gyroid with different gradient.

Figure 6 shows the compression deformation stages of Strut-Gyroid-U and Sheet-Gyroid-U at several levels of overall strain: 0%, 15%, 30%, 45%, and 60% from the video frames. The deformation process of the two types of uniform gyroid structures are similar, and almost every layer of the cellular structure is simultaneously compressed and deformed throughout the process before being densified. Besides, the stress-strain curves for the entire deformation process are shown in Figure 7a,b, and they will be discussed in the following sections along with the deformation behavior of the graded gyroid structure. The Young’s modulus of the cellular structure can be obtained from the linear elastic behavior of the stress-strain curve in the low strain region. In addition, plateau stress, densification strain, and energy absorption per volume of the cellular structures also can be obtained according to the curve. The determined values of the parameters above are recorded in Table 1. 

For the uniform structure, it can be seen from the results that the sheet-based gyroid cellular structure has a higher modulus and plastic collapse strength than the strut-based gyroid cellular structure, which is consistent with the results of numerical homogenization calculation. Moreover, the modulus and plastic collapse strength of sheet-based gyroid is around 59.4% and 33.21% larger than strut-based gyroid, respectively. Furthermore, based on the experimental results, we can also estimate the coefficients *C*_1_, *C*_2,_ and *α* of the Gibson-Ashby model, where *n* = 2 and *m* = 3/2 are assumed here. The estimation results are recorded in Table 2. For the Strut-Gyroid-U and Sheet-Gyroid-U cellular types, the determined value of *C*_1_ is in the range of 0.1 to 4.0 previously given by Gibson and Ashby [27]. However, the determined values of parameters *C*_2_ and *α* are lower than the ranges given by Gibson and Ashby from 0.25 to 0.35 and 1.4 to 2.0. This means that the plateau strength is lower than the predictable strength of the Gibson-Ashby model. Moreover, the densification strain observed here is higher than might be predicted. It is predicted that the coefficients of the Gibson-Ashby model should be calculated using cellular structures of different densities, but this is beyond the scope of this paper.

### 3.3. Deformation of Graded Gyroid-Based Cellular Structures

**Figure 8:**
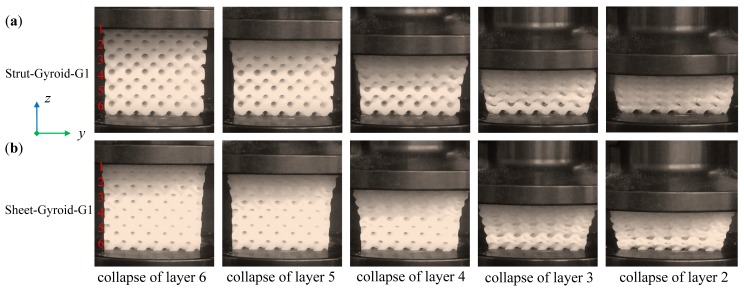
Deformation stages of layer-by-layer collapses of Strut-Gyroid-G1 (**a**) and Sheet-Gyroid-G1 (**b**) cellular structures from the video frames.

**Figure 9:**
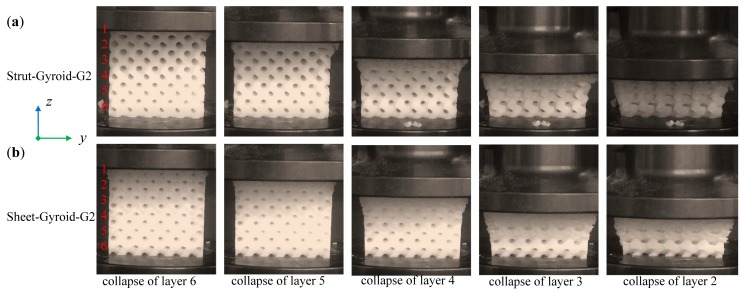
Deformation stages of layer-by-layer collapses of Strut-Gyroid-G2 (**a**) and Sheet-Gyroid-G2 (**b**) cellular structures from the video frames.

Compared with a uniform structure, the graded structures exhibit novel deformation behaviors. Figure 8 and Figure 9 show the compression deformation process of strut-based and sheet-based gyroid cellular structures under different gradients. These samples were collapsed from the initial low-density area and continuously changed layer by layer. Moreover, each layer collapses with a linear stress rise and a platform, which is a characteristic of ideal cellular solids. The above variations are also easily seen in the stress-strain curve of Figure 7. These graded models have six layers from low-density to high-density, numbered 1 to 6. The collapse behaviors of the first four layers of the Strut-Gyroid-G1 and Strut-Gyroid-G2 structures are well recognized from the video frames and stress-strain curves. However, the Sheet-Gyroid-G1 and Sheet-Gyroid-G2 structures are difficult to recognize the collapse behaviors. From a geometric point of view, it is because strut-based gyroid belongs to the cellular type of the rod-like structure, and it is prone to crush form when compressed. The sheet-based gyroid is a cellular structure formed by a more continuous surface in 3D space, so it appears to be more gradual when compressed. Moreover, it can be seen from the stress-strain diagram (in Figure 7) that the deformation behavior of the sheet-based gyroid structure is relatively smooth, while the strut-based gyroid structure has obvious collapse behavior. From the perspective of cellular anisotropy analysis, we have learned in Section 3.1 that strut-based gyroid tends to anisotropy, while sheet-based gyroid tends to be isotropic, which also can explain the stress-strain curve in Figure 7. Moreover, Young’s modulus and plastic plateau strength of gradient-1 and gradient-2 cellular structures, the sheet-based gyroid are all larger than the strut-based gyroid structures, respectively. Moreover, all the values are recorded in Table 1.

### 3.4. Energy Absorption Capability of the Cellular Structures

**Figure 10:**
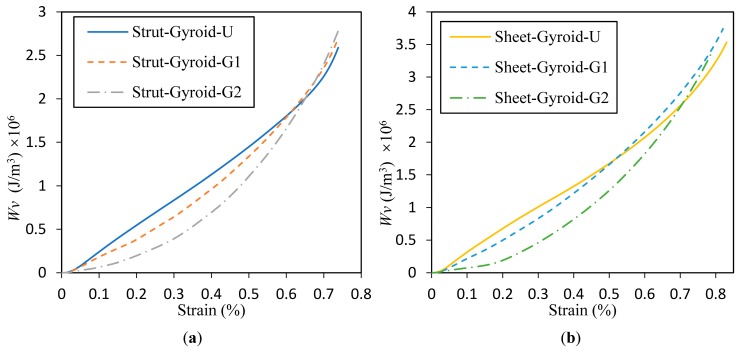
Energy absorption per unit volume for strut-based (**a**) and sheet-based (**b**) gyroid uniform and graded cellular structures.

The energy absorption capacity of the cellular structure provides an effective guide for the application of impact energy absorption [3]. The cumulative energy absorption value per unit volume *W_v_* of the cellular structure is obtained by Equation (4), that is, the integral value of the stress in the strain range is the energy absorption per unit volume when it reaches the corresponding strain value. Moreover, Figure 10 shows the relationship between strain and energy absorption value per unit volume. The total energy absorption values of the respective samples when in the densified state are recorded in Table 1. 

The curves of Strut-Gyroid-U and Sheet-Gyroid-U from Figure 10a,b have longer approximate linear regions due to the uniform cellular structure with a flat plastic strain region (about 5% to 65%). However, after the start of densification, the curves begin to deviate from their approximate linear behavior and become rapidly rising. Moreover, it can be found here that the energy absorption curve of Sheet-Gyroid-U is higher than that of Strut-Gyroid-U because the sheet-based gyroid structure of the same density has higher modulus and tends to be isotropic. From the numerical statistics of Table 1, It can be found that the densified cumulative absorbed energy of Sheet-Gyroid-U is 31.2% higher than Strut-Gyroid-U. Furthermore, the low strain region of the graded structures (Strut-Gyroid-G1, Strut-Gyroid-G2, Sheet-Gyroid-G1, and Sheet-Gyroid-G2) has significantly less energy per unit volume than the uniform structures (Strut-Gyroid-U, Sheet-Gyroid-U). However, after the weaker layers are continuously collapsed, the absorbed energy increases rapidly. For strut-based gyroid structures, the energy absorbed by Strut-Gyroid-G1 and Strut-Gyroid-G2 began to exceed Strut-Gyroid-U accordingly at approximately 60.5% and 65.4% strain, respectively. For the sheet-based gyroid, the energy absorbed by Sheet-Gyroid-G1 and Sheet-Gyroid-G2 began to exceed the Sheet-Gyroid-U accordingly at approximately 51.4% and 67.2% strain, respectively. Furthermore, the results in Table 1 shows that the densification strain of the graded structure is higher than the densification strain of the uniform structure, and the gradient-2 is higher than the densification strain of the gradient-1. It shows that the graded structure not only has higher energy absorption capacity but also provides large strain protection before densification. Moreover, Sheet-Gyroid-G2 has the highest energy absorption in all samples, which is 1.6 times the lowest energy absorption of Strut-Gyroid-U.

**Figure 11:**
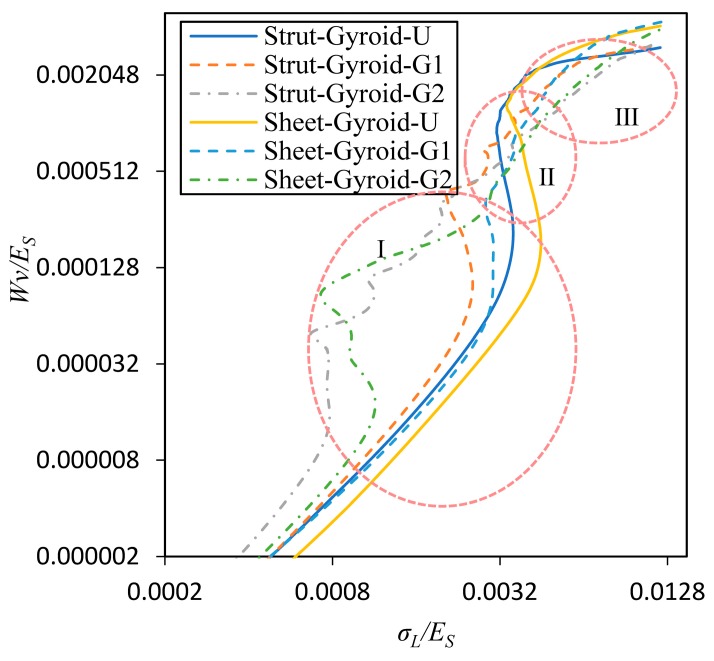
Normalized energy absorption of strut-based and sheet-based gyroid uniform and graded cellular structures.

On the other hand, in Figure 11 is demonstrated the relationship between *W_v_* and *σ_L_*, and they are normalized by the modulus of the base material, *E_S_*. This expression is useful in allowing a designer to select a cellular structure that minimizes the stress while the required energy is absorbed [27]. We divide the curve in Figure 11 into three regions, I, II, and III. First, the region I corresponds to the elastic deformation region of the uniform structure and the collapse deformation region of the low-density region of the graded structure. In this area, they all absorb less total energy. Second, in region II, the uniform structure starts to be plastically deformed, so that the absorbed energy sharply increases, and the stress increases less. However, the graded structure absorbs energy at a lower rate in this region. Third, the uniform structure enters the densified state in the III region, and a significant turning point and a subsequent rapid increase in the stress appear in the curves. In contrast, the strut-based and sheet-based gyroid structures with gradient-2 do not show a significant turning point, but gradually enters the densification state, and the rate of energy absorption generally remains unchanged. For the gradient-1 structures, there is a slight turning point, but the Strut-Gyroid-G1 is relatively more obvious than the Sheet-Gyroid-G1. Furthermore, according to the features described above, the sheet-based gyroid structure has a more gradual energy absorption process than the strut-based gyroid structure, and there is no point of sharp turning.

## 4. Conclusions

In this work, we studied the structural mechanical behavior and energy absorption capacity of the uniform and graded structure of strut-based and sheet-based gyroid in TPMS. First, the two types of gyroid structures were designed to be uniform structures and graded structures with different density intervals. Second, these samples were manufactured by SLA technology. Then, standard pressure test experiments were performed on these samples, and their stress-strain curves and values were obtained. Finally, according to the recorded experimental results, their deformation modes and energy absorption capacity were calculated. The main conclusions are as follows:

(1) Anisotropic analysis of strut-based gyroid cellular structure and sheet-based gyroid cellular structure was performed by numerical homogenization method. It is found that the sheet-based gyroid cellular structure tends to be isotropic over the entire density interval and theoretically more suited for energy absorption. In addition, the strut-based gyroid structure exhibits anisotropy, and its Young’s modulus is also smaller than the sheet-based gyroid structure at the same density.
(2) It is found from the video recorded in the experiment that the uniform structure exhibits a global collapse deformation mode during the compression process, and the graded structure exhibits a layer-by-layer collapse deformation mode from a low-density. Besides, the modulus and yield strength of the two structures are calculated according to the values of the linear elastic phase. It can be seen from the results that the modulus and the yield strength of the sheet-based gyroid structure are higher than the corresponding strut-based gyroid structure.
(3) The energy absorption per unit volume of each sample was calculated separately based on the data recorded in the experiment. It can be seen from the results that the energy absorption capacity of the graded structure is better than that of the uniform structure, and the graded structure exhibits a smoother energy absorption process until the fully dense strain. However, the uniform structure will suddenly increase sharply when it is fully dense. Additionally, the sheet-based gyroid structure has better energy absorption than the strut-based gyroid structure. Besides, the gradient levels also have an effect on energy absorption and deformation. From the results of this work, it is known that cellular structures with large gradient exhibit better energy absorption capacity than those with a small gradient.

The findings in this work indicate that different types and gradients of gyroid cellular structure can achieve different deformation behaviors and mechanical responses under compression testing. Among them, the sheet-based gyroid gradient structure is optimal in mechanical response, which can attract the application of impact-resistant vehicle or implant design. In future work, we will further study the deformation behavior and energy absorption of more gradient parameters for sheet-based graded gyroid cellular structures and try to predict their energy absorption capacity.

## Figures and Tables

**Table 1:** Mechanical properties and energy absorption of uniform and graded gyroid-based cellular structures under compressive testing.

Properties	Strut- Gyroid-U	Sheet- Gyroid-U	Strut- Gyroid-G1	Sheet- Gyroid-G1	Strut- Gyroid-G2	Sheet- Gyroid-G2
*E^H^* (MPa)	40.56	63.67	—	—	—	—
*E_L_* (MPa)	39.21 ± 0.23	62.51 ± 0.44	28.25 ± 0.31	36.67 ± 0.18	23.96 ± 0.16	32.21 ± 0.31
E* (10^−3^)	46.12 ± 0.02	73.54 ± 0.02	33.23 ± 0.05	43.14 ± 0.03	28.18 ± 0.01	37.89 ± 0.03
*σ_L_* (MPa)	2.92 ± 0.08	3.89 ± 0.12	1.98 ± 0.04	2.46 ± 0.16	0.69 ± 0.02	0.87 ± 0.05
σ* (10^−3^)	22.12 ± 0.02	29.46 ± 0.11	15.00 ± 0.12	18.63 ± 0.09	5.23 ± 0.10	6.59 ± 0.06
*ε_D_* (%)	62.3 ± 0.12	66.4 ± 0.01	68.5 ± 0.06	69.8 ± 0.13	70.1 ± 0.02	72.2 ± 0.03
*W_v_* (KJ/m^3^)	1734 ± 4	2276 ± 2	2116 ± 6	2623 ± 1	2341 ± 5	2781 ± 4

**Table 2:** Gibson-Ashby coefficients for uniform gyroid-based cellular structures.

Coefficients	*C*_1_	*C*_2_	*α*
Strut-Gyroid-U	0.512	0.135	1.257
Sheet-Gyroid-U	0.817	0.179	1.120
